# Protocol for detecting ligand-induced ubiquitination of LAG3 in human T cells

**DOI:** 10.1016/j.xpro.2025.104090

**Published:** 2025-09-13

**Authors:** Anran Dai, Xinnuo Li, Haopeng Wang, Yong Jiang

**Affiliations:** 1 Changping Laboratory, Beijing 102206, China; 2 School of Life Sciences, Central China Normal University, Wuhan 430079, China; 3 School of Life Science and Technology, ShanghaiTech University, Shanghai 201210, China

**Keywords:** Cell biology, Immunology, Molecular biology

## Abstract

Ligand-induced ubiquitination plays a critical role in regulating the immune checkpoint function of Lymphocyte Activation Gene 3 (LAG3). Here, we present a protocol for detecting LAG3 ubiquitination in T cells. We describe the establishment of a Jurkat T cell line stably expressing LAG3, followed by detailed assays to measure ligand engagement-dependent LAG3 ubiquitination using the Raji-*Staphylococcal* enterotoxin E (SEE) system as an antigen-presenting platform. This protocol provides a robust approach for investigating the molecular mechanisms underlying LAG3-mediated immune regulation through post-translational modifications.

For complete details on the use and execution of this protocol, please refer to Jiang et al.^1^

## Highlights

• Instructions for the detection of ligand-induced LAG3 ubiquitination
• Steps for generating stable LAG3-expressing human Jurkat T cells
• Procedures for ligand-receptor activation and ubiquitination studies

## Abstract (editor highlights)

Publisher’s note: Undertaking any experimental protocol requires adherence to local institutional guidelines for laboratory safety and ethics.

## Graphical abstract

**Figure:**
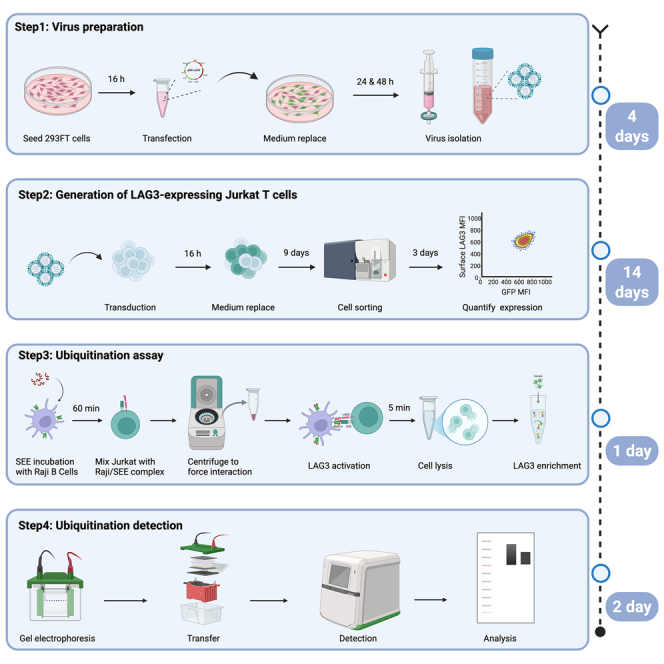

## Before you begin

Ligand-induced post-translational modifications (PTMs) are key hallmarks of immune cell activation and major regulators of immune receptor function.^2^^,^^3^ While ligand-induced ubiquitination typically acts as a negative feedback mechanism—promoting receptor degradation to prevent overactivation, as seen in FBXO38-mediated PD-1 ubiquitination^4^—our findings uncovered an unexpected positive regulatory role. Upon ligand engagement, LAG3 undergoes polyubiquitination. Critically, this modification enhances LAG3’s inhibitory function rather than acting as a negative regulator. To investigate ubiquitination of LAG3 during T cell activation, we employed a well-established T cell-antigen-presenting cell (APC) coculture system. Briefly, Jurkat T cells expressing LAG3 were cocultured with Raji B cells presenting the primary LAG3 ligand, MHC class II. The Raji cells were pulsed with superantigen to provide TCR stimulation.

Prepare all required cell lines, plasmids, and reagents prior to the experiment. The protocol below describes the specific steps for detecting ligand-induced ubiquitination of HA-tagged LAG3 in stably transfected Jurkat T cells, employing the Raji-SEE system for antigen-specific stimulation. This protocol can also be adapted to study ubiquitination of other immune receptors, such as PD-1, TIM3, and OX40, by using suitable stimulation systems and optimized experimental conditions.

### Innovation

This protocol establishes a ligand-dependent system for detecting LAG3 ubiquitination in T cells, addressing a key gap in studying its immune checkpoint regulation. Unlike non-specific stimuli or overexpression in non-native systems, this method triggers natural T cell activation pathways to specifically induce LAG3 ubiquitination. By merging these established techniques—stable cell line generation, physiological ligand presentation (Raji-SEE), and stringent ubiquitin detection—into a novel workflow, this protocol offers a significant advancement. By replacement of ligand-receptor pairs, this scalable biochemical methodology extends to studying ubiquitination dynamics of other immune receptors, offering a versatile platform for investigating post-translational signaling modulation.

### Preparation of cells

**Timing: 1 day for step 1; 4–6 days for step 2**

1. Cell thawing and recovery.
  a. Remove the cryovial from liquid nitrogen storage. Immediately submerge in a 37°C water bath. Agitate gently until ice crystals dissolve (1–2 min).
  b. Aspirate cell suspension into a 15 mL centrifuge tube with 5 mL pre-warmed complete medium (DMEM supplemented with 10% fetal bovine serum for HEK-293FT and RPMI 1640 supplemented with 5% fetal bovine serum for Jurkat and Raji).
    ***Note:*** Pre-warm the medium to 37°C before use. Add the cells slowly to the medium to avoid damage.
  c. Centrifuge the cell suspension at 300 × *g* for 3 min. Discard the supernatant.
  d. Resuspend the cells gently in 5 mL of complete medium by pipetting up and down. Plate the cells as follows:
    i. Transfer the cell suspension to a 10-cm cell culture dish for 293FT cells. Add complete medium to bring the final volume to 10 mL.
    ii. Transfer the cell suspension to a T25 cell culture flask for Jurkat and Raji cells.
  e. Place the culture vessel in a 37°C incubator with 5% CO_2_.
2. Cell passage.
  a. HEK-293FT cells (Adherent) passage.
    i. Pre-warm Trypsin-EDTA, PBS, and DMEM complete medium.
    ii. Remove the old medium. Wash the cells once with 3 mL Phosphate-Buffered Saline (PBS).
      ***Note:*** Add PBS slowly along the side wall of the culture dish to avoid dislodging adherent cells.
    iii. Add 2 mL of 0.05% Trypsin-EDTA to cover the surface. Place the dish in a 37°C incubator for 1–2 min.
    iv. When the cells become rounded, gently tap the sides of the culture dish to accelerate detachment. Immediately add 4 mL of pre-warmed complete medium to neutralize the enzyme activity.
      ***Note:*** Limit the trypsinization time (< 3 min) to avoid cell damage.
    v. Pipette the cell suspension up and down six times. Transfer the cell suspension to a 15 mL centrifuge tube.
    vi. Centrifuge the suspension at 300 × *g* for 3 min at room temperature. Discard the supernatant.
    vii. Resuspend the cell pellet in 2 mL of complete medium. Seed the cells into 10 cm dishes at a 1:6 split ratio. Add complete medium to bring the final volume to 10mL.
    viii. Place the dish in a 37°C incubator with 5% CO_2_.
  b. Jurkat and Raji cells (Suspension) passage.
    i. Gently pipette the cell suspension up and down to disperse cell clusters.
    ii. Quantify viable cells by Vi-CELL BLU Analyzer.
    iii. Transfer appropriate volume to a new flask and replenish with pre-warmed RPMI 1640 complete medium.
      **CRITICAL:** Adjust cell density to > 3×10^5^ cells/mL, concentrate the cells when density falls below 1×10^5^ cells/mL.

### Preparation of virus

**Timing: 1 day for step 3; 1 day for step 4; 2 days for step 5; 5 days for step 6**

The following protocols describe the detailed procedures for lentivirus (contain a GFP tag) package of HA-LAG3, including virus production using HEK-293FT cells and viral titer using Jurkat cells.

3. Seed HEK-293FT cells 18–24 h before transfection.
  a. Digest and collect the cells according to step 2a.
    ***Note:*** Ensure cells have undergone at least three passages after thawing, confirming they are in the logarithmic growth phase (viability > 90%).
  b. Resuspend the cells in 5 mL of antibiotic-free DMEM medium to generate a single-cell suspension.
    ***Note:*** Pipette gently until no visible clumps remain while avoiding bubble formation.
  c. Quantify viable cells by Vi-CELL BLU Analyzer.
  d. Plate 7×10^5^ cells per well in 6-well plates with a final volume of 2 mL per well. Mix gently by rocking the plate back and forth.
    **CRITICAL:** Avoid rotating the plate to prevent uneven cell distribution.
  e. Place the 6-well plate in a 37°C incubator with 5% CO_2_.
4. Plasmid DNA transfection in HEK-293FT cells.
  a. Perform transfection when cell confluence reaches 70%–80% at 18–24 h post-seeding.
  b. Add 1.05 μg of total plasmid to 250 μL of pre-warmed Opti-MEM in a 1.5 mL EP tube (as detailed in the Table 1) and then mix thoroughly by vortexing.
    **CRITICAL:** Use endotoxin-free plasmid to avoid cell cycle arrest.
  c. Add 3 μL Hieff Trans liposomal per tube at a 3:1 ratio (Hieff Trans liposomal: DNA) and flick gently to mix.
  d. Incubate for 20 min at room temperature.
  e. Carefully transfer mixture dropwise to the cells and swirl plates gently. Incubate for 6–8 h at 37°C.
  f. Remove the supernatant and slowly add 2 mL of DMEM medium (supplemented with 10% FBS and 1% PS) along the sidewall of 6-well plates.
  g. Culture cells in a 37°C incubator with 5% CO_2_.
5. Collect lentivirus.
  a. Harvest the supernatants 48 h post-transfection and store at 4°C in 15 mL centrifuge tubes.
  b. Add 2 mL of complete medium for an additional 24 h (72 h total post-transfection). Combine with the previously harvested supernatant.
  c. Filter supernatants through 0.45 μm filter to remove cell debris. Aliquot viruses into sterile tubes and store at −80°C.

***Note:*** Virus may be stored at 4°C for short periods (hours to days), but should be frozen at −80°C for long-term storage.

**Figure 1:**
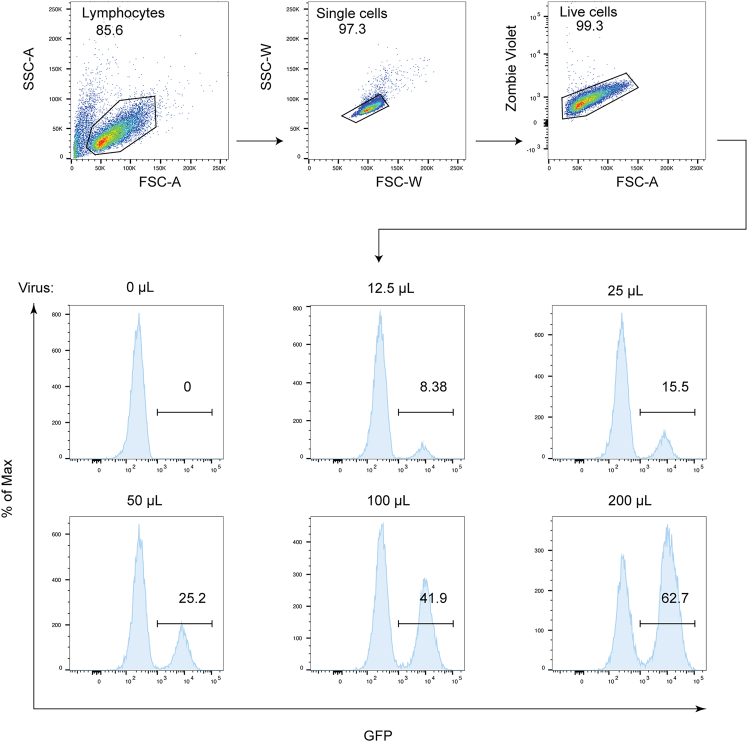
Determination of viral titer Measurement of LAG3 lentiviral transduction efficiency by Flow cytometric analysis.

6. Detect lentiviral titer (Figure 1).
  ***Note:*** This step is to determine the viral titer by flow cytometry. The method only works in cases where the lentiviral plasmid contains a fluorescent reporter gene. This protocol uses GFP as the fluorescent reporter gene. The level of fluorescence represents the titer of the virus.
  a. Adjust Jurkat cells to 1×10^6^ cells/mL and transfer 100 μL to six individual wells of a 48-well plate.
  b. Add 0, 12.5, 25, 50, 100 and 200 μL of viral stock to the wells containing Jurkat cells.
  c. Culture cells for at least 72 h in a 37°C incubator with 5% CO_2_.
    ***Note:*** Add RPMI medium to a final volume of 400 μL.
  d. Quantify viral particles by determining the percentage of GFP-positive cells in each well using flow cytometry.
    i. Transfer 200 μL of cell suspension into 96-well plates.
    ii. Centrifuge at 300g for 3 min and aspirate supernatants.
    iii. Resuspend cells in 50 μL PBS containing Zombie Violet dye (1:500 dilution).
      ***Note:*** Prepare Zombie dye working solution immediately before use and utilize within two hours.
    iv. Incubate in the dark at 4°C for 20 min.
    v. Resuspend cells in two volumes of cell staining buffer.
    vi. Centrifuge at 300g for 3 min and aspirate supernatants.
    vii. Wash two times with 200 μL cell staining buffer then resuspend in 300 μL cell staining buffer for flow cytometry (LSRFortessa X20) analysis.
  e. Calculate the number of infectious units per mL with the following formula: (of cells per well of infection GFP positive cells)×1000/(μL of virus added to well).
    ***Note:*** For accurate titer quantification, restrict analysis to samples with < 15% infection to ensure single-viral integration events. Use multi-sample titration plots for linear-range validation or single-point calculations when justified.

**Table 1:** Hieff Trans liposomal transfection system

	pCMVdR8.91(500 ng)	pMD2.G(50 ng)	HA-LAG3(500 ng)	Hieff trans liposomal
Tube1	+	+	WT	3μL
Tube2	+	+	K498R	3μL

## Key resources table

**Table:** 

REAGENT or RESOURCE	SOURCE	IDENTIFIER
**Antibodies**		

HA-Tag-HRP (6E2) (WB: 1/2,000)	Cell Signaling Technology	Cat# 3724; RRID:AB_1549585
HA-Tag (C29F4) (WB: 1/2,000)	Cell Signaling Technology	Cat# 3724; RRID:AB_1549585
Anti-Ubiquitin-HRP (P4D1) (WB: 1/2,000)	BioLegend	Cat# 646303; RRID:AB_2629597
AF647 anti-human LAG3 (3DS223H) (FC: 1/500)	Invitrogen	Cat# 51-2239-42; RRID:AB_2744740
Relatlimab	Jiang et al.^1^	N/A
HRP goat anti-rabbit IgG (H+L) (WB: 1/5,000)	Jackson ImmunoResearch	Cat# 111-035-003; RRID:AB_2313567

**Chemicals, peptides, and recombinant proteins**		

*Staphylococcal* Enterotoxin E (SEE)	Toxin Technologies	Cat# ET404
Hieff Trans liposomal transfection reagent	Yeasen	Cat# 40802ES08
N-ethylmaleimide (NEM)	Sigma-Aldrich	Cat# E3876
Protease inhibitor cocktail	Selleck	Cat# B14002
EZview Red anti-HA affinity gel	Sigma-Aldrich	Cat# E6779
6× protein loading buffer	TransGen	Cat# DL101-02
RPMI 1640	Thermo Fisher Scientific	Cat# C22400500CP
Fetal bovine serum (FBS)	Gibco	Cat# 16000-044
DMEM	Thermo Fisher Scientific	Cat# C11995500BT
Triton X-100	Sigma-Aldrich	Cat# T8787
20x MOPS SDS running buffer	ABCONE	Cat# M22785-500ML
10x Tris buffered saline with Tween 20	ABCONE	Cat# T71236-1L
Transfer buffer (10×)	Epizyme	Cat# PS109
Primary antibody dilution buffer for western blot	Epizyme	Cat# PS114
Secondary antibody dilution buffer for western blot	Epizyme	Cat# PS115
SurePAGE	GenScript	Cat# M00654
PageRuler prestained protein ladder	Thermo Scientific	Cat# 26617
SuperPico ECL master mix	Vazyme	Cat# E432-02
Cell staining buffer	4A Biotech	Cat# FXP005

**Critical commercial assays**		

Zombie Violet fixable viability kit	BioLegend	Cat# 423114
EndoFree Mini plasmid kit II	TIANGEN	Cat# DP118-02

**Experimental models: Cell lines**		

HEK-293FT (human fetal epithelial cells)	Cell Bank of Chinese Academy of Sciences	Cat#GNHu 43
Jurkat E6.1 T cells	Gifted from Arthur Weiss Lab	N/A
Raji	Gifted from Arthur Weiss Lab	N/A

**Recombinant DNA**		

pCMVdR8.91	Li et al.^5^	N/A
pMD2.G	Li et al.^5^	RRID:Addgene_12259
pHR-PGK	Morsut et al.^6^	RRID:Addgene_79120
pHR-PGK HA-LAG3	Jiang et al.^1^	N/A
pHR-PGK HA-LAG3-K498R	Jiang et al.^1^	N/A

**Software and algorithms**		

FlowJo v.10	TreeStar	https://www.flowjo.com/
ImageJ	NIH	https://imagej.nih.gov/ij/download.html

**Other**		

Amersham ImageQuant 800 (AI800)	GE Healthcare	N/A
Vi-CELL BLU	Beckman	N/A
LSRFortessa X-20	BD	N/A
Syringe driven filters	BIOFIL	Cat# FPE404030

## Materials and equipment

### Reagents for cell culture

**Table:** DMEM basic medium (for culturing 293FT cells)

Reagent	Final concentration	Amount
DMEM basic	N/A	445 mL
FBS	10%	50 mL
Penicillin/streptomycin (100×)	1 ×	5 mL
**Total**	**N/A**	**500 mL**

Store at 4°C for up to 3 weeks.

**Table:** RPMI 1640 basic medium (for culturing Jurkat and Raji cells)

Reagent	Final concentration	Amount
RPMI 1640 basic	N/A	470 mL
FBS	5%	25 mL
Penicillin/streptomycin (100×)	1×	5 mL
**Total**	**N/A**	**500 mL**

Store at 4°C for up to 3 weeks.

**Table:** MACS buffer (for cell sorting)

Reagent	Final concentration	Amount
PBS	N/A	48.8 mL
FBS	2%	1 mL
0.5 M EDTA (pH 8.0)	2 mM	0.2 mL
**Total**	**N/A**	**50 mL**

Store at 4°C for up to 3 weeks and sterilize by filtration through a 0.2-μm filter.

### Reagents for ubiquitination assay

**Table:** 2 M NaCl solution

Reagent	Final concentration	Amount
NaCl	2 M	58.5 g
H_2_O	N/A	Up to 500 mL
**Total**	**N/A**	**500 mL**

Store at room temperature for up to 1 year.

**Table:** 1 M N-ethylmaleimide solution

Reagent	Final concentration	Amount
N-ethylmaleimide	1 M	1.25 g
Ethanol absolute	N/A	Up to 10 mL
**Total**	**N/A**	**10 mL**

Store at −20°C protected from light for up to 6 months. Avoid multiple freeze-thaw cycles by storing samples in aliquots.

**CRITICAL:** NEM poses acute irritant and corrosive hazards via inhalation or dermal exposure. Operators must wear protective equipment such as nitrile gloves, goggles, and a face mask while working inside a fume hood.

**Table:** 10% SDS solution

Reagent	Final concentration	Amount
SDS	10% (w/v)	5 g
H_2_O	N/A	Up to 50 mL
**Total**	**N/A**	**50 mL**

Store at room temperature for up to 1 year.

**CRITICAL:** SDS powder is irritating and corrosive. Inhalation causes respiratory damage, while skin or eye contact leads to chemical corrosion injuries. Operate strictly inside a fume hood while wearing a face mask and nitrile gloves. During dissolution, always add the powder slowly to water (reverse addition is strictly forbidden).

**Table:** 2× buffer A

Reagent	Final concentration	Amount
2 M NaCl solution	300 mM	75 mL
1 M Tris-HCl (pH 7.5)	100 mM	50 mL
Triton X-100	2% (v/v)	10 mL
H_2_O	N/A	365 mL
**Total**	**N/A**	**500 mL**

Store at 4°C for up to 6 months.

**Table:** 2× lysis buffer

Reagent	Final concentration	Amount
2× Buffer A	N/A	47 mL
10% SDS solution	0.2%	1 mL
1 M N-ethylmaleimide solution	20 mM	1 mL
Protease Inhibitor Cocktail (100×)	2×	1 mL
**Total**	**N/A**	**50 mL**

Prepare the solution just before use.

**Table:** 1× wash buffer

Reagent	Final concentration	Amount
2× Buffer A	1×	25 mL
10% SDS solution	0.1%	0.5 mL
H_2_O	N/A	25 mL
**Total**	**N/A**	**50 mL**

Prepare the solution just before use.

### Reagents for western blot

**Table:** 1× transfer buffer

Reagent	Final concentration	Amount
Transfer buffer (10×)	1×	100 mL
Methanol	N/A	200 mL
H_2_O	N/A	700 mL
**Total**	**N/A**	**1 L**

Store at 4°C for up to 2 weeks. The 10× transfer buffer must be diluted with deionized water prior to methanol addition.

**CRITICAL:** Methanol is highly volatile, flammable, and extremely toxic. All operations must be performed in a fume hood while wearing nitrile gloves, safety goggles, and a face mask to prevent skin contact or inhalation.

**Table:** Blocking buffer

Reagent	Final concentration	Amount
Non-fat milk	5% (w/v)	1 g
TBST (1×)	N/A	Up to 20 mL
**Total**	**N/A**	**20 mL**

Store at 4°C for up to 3 days.

## Step-by-step method details

### Generation of a stable LAG3-expressing Jurkat cell line

**Timing: 10 days for step 1; 4 days for step 2**

This step describes the generation of stable LAG3-expressing Jurkat cells through a lentiviral vector system expressing GFP tag, followed by enrichment of LAG3^+^ populations via fluorescence-activated cell sorting (FACS) and validation of constitutive protein expression. Since the lysine 498 residue serves as the sole ubiquitination site on LAG3, we generated an LAG3-K498R Jurkat cell line as a negative control for ubiquitination assays.

**Figure 2:**
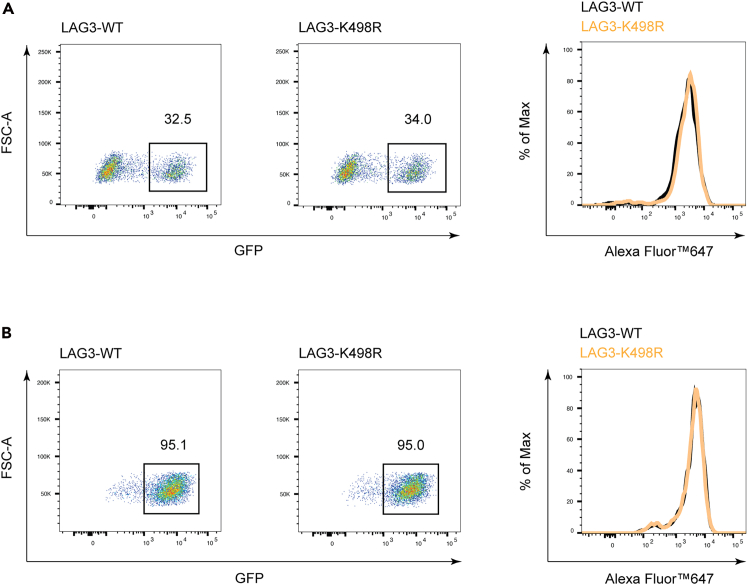
Generation of a stable LAG3-expression Jurkat cell line (A and B) Quantify the percentage of LAG3^+^ cells and surface expression of LAG3 before(A) and after (B) sorting.

1. Transduction of LAG3 lentivirus.
  a. Adjust Jurkat cells to 1×10^6^ cells/mL and transfer 100 μL to two individual wells of a 48-well plate.
  b. Add lentiviral particles expressing LAG3-WT and LAG3-K498R into individual wells of a 48-well plate.
  c. Culture cells for 16 h in a 37°C incubator with 5% CO_2_.
  d. Remove most of the supernatant and slowly add 500 μL of RPMI 1640 complete medium to two individual wells of a 48-well plate.
  e. After two days, harvest a small aliquot of cells for flow cytometry to determine the percentage of LAG3^+^ cells and median fluorescence intensity (MFI) of LAG3 (see troubleshooting 1 and 2) (Figure 2A).
    i. Transfer 100 μL of cell suspension into 96-well plates. Centrifuge at 300 × *g* for 3 min and aspirate supernatants.
    ii. Resuspend cells in 50 μL PBS containing Zombie Violet dye (1:500 dilution).
      ***Note:*** Prepare zombie dye working solution immediately before use and utilize within two hours.
      **CRITICAL:** Don’t use Tris buffer as a diluent and be sure that the PBS does not contain any other protein like BSA or FBS. For cell types sensitive to protein-free conditions, titrate Zombie Violet dye with BSA/serum matching the antibody staining buffer. Increase dye dosage to compensate for BSA/serum binding.
    iii. Incubate in the dark at 4°C for 20 min.
    iv. Wash one time with 200 μL cell staining buffer.
    v. Resuspend cells in 50 μL cell staining buffer containing Alexa Fluor647 anti-hLAG3 antibody (1:500 dilution).
      ***Note:*** Prepare fluorochrome-coupled antibody working solution just before use.
    vi. Incubate in the dark at 4°C for 20 min.
    vii. Centrifuge at 300 × *g* for 3 min and aspirate supernatants.
    viii. Wash three times with 200 μL cell staining buffer then resuspend in 300 μL cell staining buffer for flow cytometry analysis.
  f. Culture cells in a 37°C incubator with 5% CO_2_ and expand cells to 5×10^6^ cells for subsequent isolation of LAG3^+^ populations by FACS (see troubleshooting 3).
    ***Note:*** Adjust cell density to optimal levels by adding fresh medium when density exceeds 2×10^6^ cells/mL.
2. Isolation of LAG3-expressing Jurkat cells by FACS.
  a. Harvest the majority of cells and centrifuge at 300 × *g* for 3 min to remove supernatant.
    ***Note:*** Maintain the remaining cells in small-scale culture as a backup to mitigate risks during flow cytometry sorting—such as instrument failure or mycoplasma contamination—ensuring alternative cell reserves are available.
  b. Resuspend the cells in 1 mL MACS buffer and transfer to a 5 mL flow tube with cell strainer cap.
    **CRITICAL:** Cell filtration through 40 μm strainers removes aggregates, preventing flow cytometer clogging.
  c. Isolate approximately 0.5 million LAG3^+^ cells by FACS.
  d. Resuspend cells in complete medium and seed into a 24-well plate for expansion culture (see troubleshooting 4).
  e. After approximately three days of sorting, harvest a small aliquot of cells for flow cytometry to determine the percentage of LAG3^+^ cells and median fluorescence intensity (MFI) of LAG3 (same step as shown in the step 1e) (Figure 2B).
  f. Subsequently, scale up the culture for ubiquitination assay.

### Assay of ligand-triggered LAG3 ubiquitination

**Timing: 1 day for steps 3–4; 2 days for step 5**

Ubiquitination of LAG3 requires simultaneous T cell receptor (TCR) activation and ligand engagement. Therefore, this step utilizes an established T cell-APC co-culture system^7^^,^^8^ (Raji-SEE (Staphylococcal Enterotoxin E)) to induce LAG3 ubiquitination, followed by detection via immunoblotting. LAG3-K498R mutant (ubiquitination-defective) and anti-LAG3 blocking antibody (ligand-engagement defective) are utilized as controls respectively in this section.

3. Ligand-induced LAG3 ubiquitination.
  a. For the LAG3-K498R (ubiquitination-defective) mutant serving as the control group, perform the following treatments (as detailed in the Table 2):
    i. Harvest LAG3-WT and LAG3-K498R Jurkat cells, respectively, followed by centrifugation to remove supernatant.
      ***Note:*** Typically, ∼10 million Jurkat cells are used per sample.
    ii. Resuspend the cells in serum-free RPMI 1640 medium at a density of 1×10^7^ cells/mL.
    iii. Incubate at 37°C for 30 min to induce serum starvation.
      ***Note:*** This starvation is critical for resetting the activation threshold of TCR signaling pathways, thereby minimizing noise in signal detection assays.
  b. For the anti-LAG3 blocking antibody (ligand-engagement defective) serving as the control group, perform the following treatments (as detailed in the Table 3):
    i. Harvest LAG3-WT Jurkat cells, followed by centrifugation to remove supernatant.
    ii. Resuspend the cells in serum-free RPMI 1640 medium at a density of 1×10^7^ cells/mL.
    iii. Incubate at 37°C for 30 min to induce serum starvation.
    iv. Divide the cell suspension into two equal aliquots. To one aliquot, add LAG3 blocking antibody to a final concentration of 10 μg/mL. To the other aliquot, add an equal volume of PBS.
    v. Incubate on ice for 30 min.
  c. Calculate the required number of Raji cells based on a Jurkat: Raji ratio of 2:1, then resuspend Raji cells in serum-free RPMI 1640 medium to a final density of 1.5×10^7^ cells/mL.
  d. Pre-incubate Raji cells with 50 ng/mL SEE for 1 h at 37°C.
    ***Note:*** This step ensures sufficient binding between MHC class II molecules on Raji cells and SEE superantigens.
    ***Note:*** The processed Raji cells from this step can be directly used in subsequent procedures without centrifugation.
  e. Place the pre-treated Jurkat and Raji cells on ice after treatment.
  f. Centrifuge Jurkat cells and discard the supernatant.
  g. Resuspend the pellet in pre-cooled serum-free RPMI 1640 medium to a density of 3×10^7^ cells/mL.
  h. Transfer 300 μL aliquots of Jurkat cell suspension and 300 μL aliquots of Raji cell suspension into the same microcentrifuge tube maintained on ice, combining them into a single 600 μL mixture for each time-point sample.
  i. Mix thoroughly by pipetting 3 times, then centrifuge at 600 × *g* for 1 min at 4°C.
    ***Note:*** Centrifuge the cells at 4°C to promote intercellular contact. Maintain this low- temperature condition to prevent premature cell activation during centrifugation.
  j. Immediately transfer the centrifuged sample to a 37°C water bath for activation.
  k. For 0-min samples, directly add 600 μL of ice-cold 2× Lysis buffer to terminate. For other samples, immediately add 600 μL of ice-cold 2× Lysis buffer for termination upon reaching the indicated time point (as detailed in the Tables 2 or 3).
    ***Note:*** Resuspend cell pellet thoroughly when adding 2× Lysis buffer to ensure complete termination.
4. Enrichment of HA-tagged LAG3 protein.
  a. Incubate the samples for 1 h at 4°C with constant rotation for lysis (see troubleshooting 5).
  b. Centrifuge at 12,000 × *g* for 15 min at 4°C to pellet cell debris.
  c. Transfer 100 μL of lysate supernatant from step 4b to a 1.5 mL microcentrifuge tube. Add 20 μL 6× Loading buffer to prepare the input samples.
  d. Collect 1 mL of lysate supernatant from step 4b and add 15 μL of EZview Red Anti-HA Affinity Gel beads. Incubate at 4°C for 1 h with rotating mixer.
  e. Centrifuge at 8,200 × *g* for 30 s at 4°C and carefully discard the supernatant without disturbing the pellet.
  f. Wash the bead pellet by adding 1 mL of 1× Wash buffer. Incubate at 4°C for 3 min with rotating mixer.
  g. Centrifuge at 8,200 × *g* for 30 s at 4°C and carefully discard the supernatant without disturbing the pellet.
  h. Repeat washes two more times as in step 4f–4g.
  i. Add 100 μL of 2× Loading buffer to the beads and mix gently to prepare the IP samples.

**Pause point:** Store the input and IP samples at −20°C until use in SDS-PAGE.

5. Detection of LAG3 ubiquitination by immunoblotting.
  a. Gel electrophoresis.
    i. Boil input and IP samples for 5 min. Centrifuge 30 s at 8,200 × *g* to pellet beads.
    ii. Run 20 μL of the supernatant on a SurePAGE gel (4–12%) at 90 V for 20 min, then switch to 120 V for 1 h.
  b. Transfer.
    i. Pre-wet PVDF membrane in methanol for 1–2 min.
    ii. Equilibrate the PVDF membranes and the gels in the transfer buffer for 5 min.
    iii. Assemble transfer sandwich and transfer at 200 mA for 1 h.
      ***Note:*** Methanol can be replaced with absolute ethanol and the transfer buffer should be adjusted to include ethanol.
  c. Blocking.
    i. Wash the PVDF membrane once with TBST (Tris-buffered saline with 0.1% Tween-20).
    ii. Block with 5% non-fat milk in TBST at room temperature for 1 h.
    iii. Wash 3 times with TBST for 5 min each time after blocking.
  d. Antibody Incubation.
    i. Incubate with HRP-conjugated primary antibody diluted in 10 mL of Secondary Antibody Dilution Buffer on a shaker at 4°C overnight.
      ***Alternatives:*** PVDF membranes can be incubated on a shaker at 20°C–25°C for 1 h.
      ***Alternatives:*** PVDF membranes can also be incubated with primary antibody (diluted in Primary Antibody Dilution Buffer) followed by species-matched secondary antibody (diluted in 5% non-fat milk).
    ii. The next day, wash 3 times with TBST for 5 min each time.
  e. Detection.
    i. Prepare ECL substrate immediately before use.
    ii. Apply ECL substrate evenly to the membranes.
    iii. Capture chemiluminescent signals using an imaging system (Amersham ImageQuant 800) (see troubleshooting 6).

**Table 2:** LAG3 ubiquitination system I

Time point (min)	0	1	2	5	15	30
SEE (ng/mL)	50 ng/mL					
LAG3-WT	∼10 million per sample					
LAG3-K498R	∼10 million per sample					
Raji	∼5 million per sample

**Table 3:** LAG3 ubiquitination system II

Time point (min)	0	1	2	5	10	15	30
SEE (ng/mL)	50 ng/mL						
Treatment	Relatlimab (10 μg/mL) or PBS (0.1 mol/L)						
LAG3-WT	∼10 million per sample						
Raji	∼5 million per sample						

## Expected outcomes

**Figure 3:**
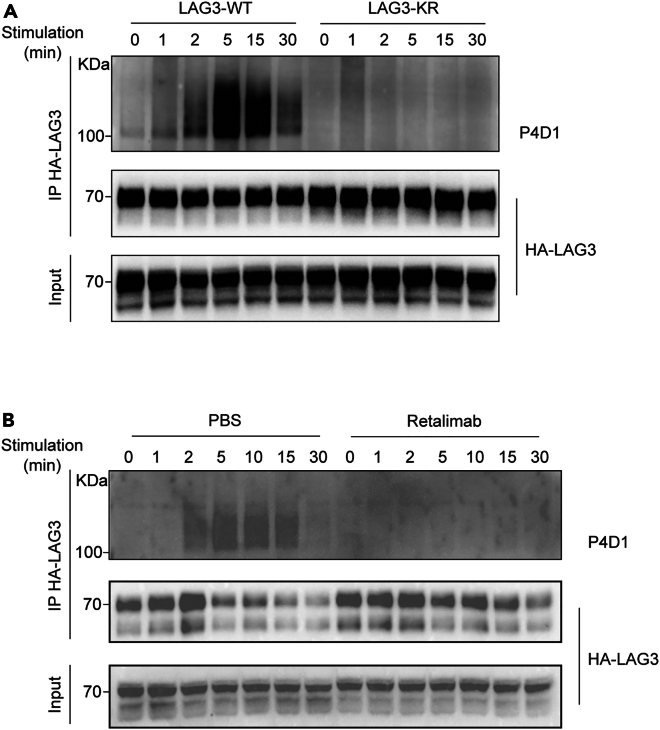
Ligand-induced LAG3 ubiquitination (A and B) LAG3 ubiquitination kinetics. LAG3-K498R mutant (ubiquitination-defective) (A) and anti-LAG3 blocking antibody (ligand-engagement defective) (B) are utilized as controls respectively.

The ubiquitination assay is expected to demonstrate rapid and transient ubiquitination of LAG3 following antigen stimulation (Figure 3). Upon LAG3 stimulation, ubiquitination should be detectable as early as 2 min, with signal intensity peaking at 5 min before gradually declining. By 30 min, ubiquitination levels are anticipated to return nearly to baseline. Unstimulated LAG3 (0 min) should show minimal ubiquitination, serving as a baseline reference. No ubiquitination signal is detectable in the control samples (LAG3-K498R mutant and anti-LAG3 blocking antibodies).

## Limitations

This protocol optimized for detecting LAG3 polyubiquitination in T cells, which requires modification for application in other LAG3-expressing cell types such as NK cells. A current limitation of this approach is the inability to evaluate ubiquitination at single-cell resolution by flow cytometry, owing to the lack of specific antibodies against ubiquitinated LAG3. Furthermore, the absence of identified deubiquitinating enzymes (DUBs) targeting LAG3 precludes experimental validation of the precise regulatory mechanisms controlling LAG3 ubiquitination.

## Troubleshooting

### Problem 1

Low lentivirus transduction efficiency (step 1e of step-by-step method details).

### Potential solution

• Check the concentration of the plasmid before transfection.
• Verify the status of 293FT cells.
• Examine the fluorescence of 293FT cells after transfection.
• Use fresh virus and avoid repeated freeze-thaw cycles.

### Problem 2

Inconsistent expression levels of LAG3 wild-type and mutants (step 1e of step-by-step method details).

### Potential solution

Standardize the viral infection level to ensure consistency (Figure 1).

### Problem 3

Poor viability of the Jurkat T cells post-transduction (step 1f of step-by-step method details).

### Potential solution

Control the viral infection efficiency to below 50% (Figure 2A).

### Problem 4

Poor viability of the Jurkat T cells after cell sorting (step 2d of step-by-step method details).

### Potential solution

• Perform cell sorting in RPMI 1640 medium supplemented with 5% fetal bovine serum.
• Detect whether there is mycoplasma contamination.

### Problem 5

Inadequate cell lysis (step 4a of step-by-step method details).

### Potential solution

• Extend the lysis time.
• Vortex the mixture every 10 min.

### Problem 6

Weak ubiquitination signal (step 5e of step-by-step method details).

### Potential solution

• Check the surface-LAG3 expression level on Jurkat cells.
• Proportionally increase the cell quantity in the reaction system.
• Optimize stimulation time to 5–10 min (avoid < 5 min or > 15 min).
• Use more beads to enrich LAG3 protein.
• Verify whether NEM is added to the cell lysis buffer.
• Prepare fresh antibodies for Western Blot detection.
• Employ hypersensitive developer for film exposure.

## Resource availability

### Lead contact

Further information and requests for resources and reagents should be directed to and will be fulfilled by the lead contact, Yong Jiang (jycpl@cpl.ac.cn).

### Technical contact

Questions about the technical specifics of performing the protocol should be directed to and will be answered by the technical contact, Anran Dai (dar@cpl.ac.cn).

### Materials availability

Materials are available upon reasonable request.

### Data and code availability

This paper does not report original code.

## Acknowledgments

This work was supported by grants from Changping Laboratory, the National Natural Science Fund Original Exploratory Program (82350110), the STI 2030-Major Project (2023ZD0520200), the Program of Shanghai Academic Research Leader, the Shanghai Frontiers Science Center for Biomacromolecules and Precision Medicine at ShanghaiTech University, the Central Guidance on Local Science and Technology Development Fund (YDZX20233100001002 and YDZX20223100001002), and the Shanghai Local College Capacity Building Project (22010502700). Y.J. was funded by a grant from the Shanghai Super Postdoctoral Incentive Program. The graphical abstract was created with BioRender.com.

## Author contributions

A.D. and Y.J. developed and optimized the methods. A.D., X.L., H.W., and Y.J. wrote the manuscript.

## Declaration of interests

The authors declare no competing interests.
